# Therapeutic effects of melittin on paclitaxel-induced peripheral neuropathic pain and spinal neuronal hyperactivity in male rats

**DOI:** 10.3389/fnins.2025.1622553

**Published:** 2025-07-02

**Authors:** Daxian Li, Yan Wu, Lirong Chang, Yizhi Song, Xiaoqiang Du, Fenqin Xue, Yang Liu, Jie Wu, Tianlong Wang

**Affiliations:** ^1^Department of Anesthesiology, Xuanwu Hospital, Capital Medical University, Beijing, China; ^2^National Clinical Research Center for Geriatric Diseases, Beijing, China; ^3^Beijing Key Laboratory of Neural Regeneration and Repair, Department of Anatomy, School of Basic Medical Sciences, Beijing Institute of Brain Disorders, Capital Medical University, Beijing, China; ^4^College of Veterinary Medicine, Beijing University of Agriculture, Beijing, China; ^5^Core Facilities Center, Capital Medical University, Beijing, China

**Keywords:** chemotherapy-induced peripheral neuropathy, bee venom, acupuncture, analgesia, wide dynamic range neuron, electrophysiological recording, noradrenergic system

## Abstract

**Introduction:**

As a taxane-based cytostatic agent, paclitaxel holds a broad spectrum of life-saving properties. However, its use is frequently limited by painful neuropathy in the extremities, which severely hinders the ultimate prognosis of cancer survivors. While bee venom therapy has shown promise in alleviating chemotherapy-induced neuropathic pain, the analgesic potential of its primary bioactive components, such as melittin and phospholipase A2 (bvPLA2), remains uncharacterized. This study investigated the ameliorative effects of melittin against paclitaxel-induced peripheral neuropathy in rats through integrated behavioral, *in vivo* electrophysiological, and neuropharmacological approaches.

**Methods:**

Paclitaxel was administered intraperitoneally (i.p.) at a total dose of 8 mg/kg. Cold and mechanical allodynia and hyperalgesia were quantified using the acetone drop and von Frey filament tests. To compare the therapeutic properties of bee venom ingredients, either melittin (0.5 mg/kg) or bvPLA2 (0.12 mg/kg) was administered subcutaneously at ST36 (Zusanli acupoint). *In vivo* extracellular recordings of wide dynamic range (WDR) neurons were performed in the spinal dorsal horn. Noradrenaline depletion was induced by the i.p. treatment with N-(2-Chloroethyl)-N-ethyl-2-bromobenzylamine (DSP-4, 50 mg/kg), and serotonin depletion was conducted by the i.p. administration of para-chlorophenylalanine (PCPA, 450 mg/kg).

**Results:**

ST36 treatment with melittin, but not bvPLA2, markedly impeded mechanical and cold hypersensitivity. Electrophysiological analysis revealed that paclitaxel induced spontaneous and stimulus-evoked hyperexcitation of spinal WDR neurons. Melittin selectively suppressed evoked neuronal activities (i.e., acute responses and after-discharges) without modulating the spontaneous firing of WDR neurons. Neuropharmacological investigation demonstrated that the effects of melittin were fully reversed by noradrenaline depletion, whereas serotonin depletion had no effect.

**Discussion:**

Our findings establish that melittin treatment at ST36 could ease paclitaxel-induced neuropathic pain by partially attenuating the hyperexcitable state of spinal WDR neurons. Furthermore, these ameliorative actions were mediated by the specific recruitment of the endogenous noradrenergic system. This study provides novel evidence supporting melittin as a targeted symptomatic agent for paclitaxel-induced peripheral neuropathy, which would advance the development of promising analgesic strategies in oncological care.

## 1 Introduction

Paclitaxel, a taxoid extracted from the Pacific yew tree, is the mainstay of gynecologic, lung, and pancreatic cancer treatments (Tsai et al., 2021; Yu et al., 2022). Apart from the potent cytostatic impact on tumor cells, paclitaxel is often accompanied by non-negligible ongoing or evoked neuropathic complications, alongside serious sensory dullness in the digits, which can finally compromise the life-saving antineoplastic therapy (Hershman et al., 2014; Cavaletti and Marmiroli, 2015). Nearly as high as 60–70% of patients develop untoward dose-dependent acute or chronic neuropathy, ranging from the early phases of chemotherapy to even years after the last prescription of paclitaxel (Polomano et al., 2001; Boyette-Davis et al., 2015). Due to the global aging population and advancements in tumor diagnostics, chemotherapy-induced peripheral neuropathy (CIPN) is likely to become increasingly common and ultimately pose an escalating socioeconomic burden (Yu et al., 2022). Though prevailing painkillers, such as opiates and NSAIDs, are still empirically prescribed for the relief of CIPN, their overall benefits appear to remain under debate (Marupudi et al., 2007; Sisignano et al., 2014; Boyette-Davis et al., 2015). Regardless of intensified breakthroughs, up until now, no clinically identified symptomatic or prophylactic schemes have been recommended for addressing paclitaxel-induced neuropathic symptoms (Marupudi et al., 2007).

*Apipuncture*, or bee venom acupuncture (BVA), has potent analgesic activities in traditional oriental and western medical systems (Zhang et al., 2018; Sung and Lee, 2021; Stela et al., 2024). Advances in the methodologies of extraction and isolation of bee venom are stimulating the exploration of its analgesic characteristics (Kwon et al., 2001; Lee et al., 2001; Lin and Hsieh, 2020). The analgesic potential of acupuncture with bee venom and its main bioactive constituents, such as melittin and phospholipase A2 (PLA2), has been studied in a spectrum of painful states, including central post-stroke pain, inflammatory pain, osteoarthritis, and neuropathic pain, among others (Park et al., 2012; Yoon et al., 2012; Seo et al., 2017; Zhang et al., 2018; Choi et al., 2019; Woo et al., 2019; Li et al., 2020a,b; Lin and Hsieh, 2020). However, the central sensitization of CIPN is a complex process (Millan, 2002; Boyette-Davis et al., 2015, 2018; Shim et al., 2019; Zajaczkowska et al., 2019), and the spinal neuronal modulation of apitherapy in paclitaxel-induced neuropathic pain is poorly understood. We previously found that 1.0 mg/kg of BVA at ST36 (Zusanli acupoint) displayed anti-allodynic properties in neuropathic rodents receiving paclitaxel (Li et al., 2020b). Our subsequent *in vivo* single-unit recording research implied that BVA ameliorated excessive firing events of wide dynamic range (WDR) neurons in the spinal dorsal horn within vincristine-induced neuropathic states (Li et al., 2020a). Anti-allodynic effects of bee venom contained therapy could be modulated by activations of the noradrenergic or serotonergic system in paclitaxel-induced neuropathy (Li et al., 2020b). Nevertheless, the effects of acupuncture with melittin or bvPLA2 on hyperexcitation of spinal dorsal horn pain-relay neurons remain undefined. Furthermore, the potential analgesic mechanism of the primary components of bee venom within paclitaxel-induced neuropathic pain is poorly characterized. Deciphering these mechanisms is crucial for developing alternative strategies based on bee venom to manage neuropathic pain.

In this paper, we initially probed whether pharmacoacupuncture of melittin or bvPLA2 at ST36 could ease paclitaxel-induced painful comorbidities in rats. Secondly, utilizing *in vivo* electrophysiological recordings of the dorsal horn WDR neurons, we gauged spinal spontaneous and stimulus-evoked signals within neuropathic states and their suppressive properties after ST36 peripheral treatments. Finally, by irreversible depletion of specific neurotransmitters in rats, we dissected whether the endogenous serotonergic or noradrenergic inhibitory system could make a key contribution to the analgesic potential of bee venom-based therapy.

## 2 Materials and methods

### 2.1 Animal preparation

In this study, the analgesic efficacy and mechanisms of apitherapy were evaluated in a series of randomized double-blind trials involving 137 male Sprague-Dawley (SD) rats. Concisely, SD rats, aged 5 weeks postnatal, were purchased (160–180g; SPF grade; Vital River Laboratory Animal Technology Co., Ltd., Beijing, China) and reared with unrestricted access to chow and water (*n* = 3/cage). The breeding room was kept at 22 ± 1°C under an artificial 12-h light-dark cycle (light cycle: 8:00 AM to 8:00 PM).

In the *in vivo* spinal recording investigation, n refers to the number of spinal WDR neurons, while in other trials, *n* refers to the number of animals. Based on previous studies using chemotherapy-induced neuropathic pain models in male rodents, a sample size of 8 to 12 rats per group was selected for the behavioral assay. For the extracellular single-unit recording and the neuropharmacological test, sample sizes were set at 12 to 13 neurons per group (each single-unit recording obtained per trial) and 6 to 7 rats per group, respectively (Li et al., 2015; Chae et al., 2019; Choi et al., 2019; Li et al., 2020a,b; Kim et al., 2023). Accordingly, animal usage was as follows: *n* = 47 in behavioral assessment, *n* = 49 in extracellular recording, and *n* = 41 in neuropharmacological test. Rats were randomly allocated to each group (see figure legends for treatment-specific sample sizes), and the investigator conducting behavioral and spinal recordings was blinded to the drugs and interventions. Animal research protocols were ratified by the Ethical Committee of Capital Medical University (Nos. AEEI-2023-036; approved in March 2023, and AEEI-2024-119; approved in May 2024) and administered following the ethical guidelines of the International Association for the Study of Pain (IASP) (Zimmermann, 1983).

### 2.2 Behavioral assessment

The rats were fully acquainted with the experimental circumstances and familiarized with the investigator 1 week before the behavioral trial (days 1 to 7) (Li et al., 2019, 2021). Each animal was enclosed beneath an inverted transparent plastic cage (15 × 19 × 28 cm) atop a stainless-steel mesh floor, and left to acclimate for 30 min before the evaluation (Li et al., 2020a).

To identify peripheral cold allodynia, the brisk hind paw flicking and licking frequency evoked by cutaneous acetone stimuli was quantified over 30 s (Yoon et al., 1994; Li et al., 2019). Using a pipette fitted with polyethylene tubing at the front, 50 μL of acetone was applied topically to the ventral surface of the right hind paws three times at 10-min intervals, and the total responses were averaged per set (Li et al., 2020a).

Peripheral mechanical allodynia and hyperalgesia assays were conducted according to established methods (Flatters and Bennett, 2004; Zheng et al., 2011; Li et al., 2019). Each von Frey Filament (VFF, RWD Life Science Co., Ltd, Sugar Land, TX, USA) stimulus was perpendicularly applied to the mid-plantar surface of the right hind limb 10 times, once every 10 s. Rapid withdrawal frequencies of the hind paw elicited by calibrated filaments (4 g for innocuous and 15 g for noxious stimulation in adult rats) were evaluated and calculated as the total percentage reaction (Li et al., 2020a). Peripheral hypersensitivities were identified by stimulating the hind paw in the following order: a von Frey filament with bending forces of 4 g and 15 g, and acetone drops.

### 2.3 Paclitaxel regimen

To establish cold and mechanical CIPN, the chemotherapeutic group received four intraperitoneal administrations of paclitaxel on alternating days (i.p., 2 mg/kg/day; days 8, 10, 12, and 14; Macklin Biochemical Technology Co., Ltd., Shanghai, China) (Polomano et al., 2001; Li et al., 2020b; Lee et al., 2022; Kim et al., 2023). Paclitaxel was dissolved at 6 mg/mL with a mixture of Cremophor EL (Macklin Bio-chemical Technology Co., Ltd., Shanghai, China) and absolute ethanol half in each, further diluted with normal saline (SAL; China Resources Double-Crane Pharmaceutical Co., Ltd., Beijing, China) at a final concentration of 2 mg/mL (Li et al., 2020b). For comparison, an equivalent volume of vehicles (ethanol: Cremophor EL: saline, 1:1:4) was treated as the controls.

### 2.4 Melittin and bvPLA2 treatments at ST36

Melittin (a peptide; Macklin Biochemical Technology Co., Ltd., Shanghai, China) and bvPLA2 (an enzyme; Sigma, St. Louis, MO, USA) were dissolved in 50 μL of SAL, and subcutaneously (s.c.) administered into the right hind limb ST36 with a dose of 0.5 and 0.12 mg/kg, respectively (Li et al., 2020a). The formula of apitherapy was tailored to literature data showing analgesic efficacies without adverse events in rats (Choi et al., 2019; Li et al., 2020a). In adult rats, ST36 is located at the tibialis anterior muscle, 5 mm lateral and distal to the anterior tibial tubercle (Yin et al., 2008; Choi et al., 2019; Li et al., 2020a).

### 2.5 *In vivo* spinal extracellular recording

Single-unit recordings of WDR neurons were performed in the spinal dorsal horn (Kim et al., 2017; Yamada et al., 2018; Chae et al., 2019; Choi et al., 2019). In brief, rats were stably anesthetized with urethane (0.24 g/mL, 1.5 g/kg, i.p.; Sigma, St. Louis, MO, USA), followed by thoracolumbar vertebral laminectomy to expose the dorsal surface of the spinal L3 to L5 regions under conditions of complete loss of withdrawal reflex (Sikandar et al., 2017; Li et al., 2020a). To fasten in a stereotaxic frame (Narishige, Tokyo, Japan) in a prone position, two rostral clamps stabilized the T12 and L3 vertebrae, respectively. Simultaneously, with a superfusion of Krebs solution (Hickey et al., 2014), the dura mater was smoothly peeled. At a 30-degree angle, an insulated tungsten electrode (resistance 10 MΩ; L504-0181, FHC, Bowdoin, ME, USA) was placed into the right dorsal horn of the exposed lumbar enlargement (Choi et al., 2017). After that, to identify the peripheral receptive field corresponding to the isolated WDR neuron, cutaneous brush, pinch, and acetone cooling stimuli were sequentially applied to the ventral skin of the ipsilateral hind paw (Kim et al., 2017; Chae et al., 2019; Choi et al., 2019; Li et al., 2020a). Each 4-s brush stimulus was performed by stroking the skin five times using a camel brush (Kim et al., 2017). A pinch stimulus was given by pinching the receptive field for 4 s with toothed forceps (Fine Science Tools, Heidelberg, Germany) (Sikandar et al., 2017; Choi et al., 2019; Li et al., 2020a). An innocuous evaporative cold stimulus was administered by applying 50 μL of acetone (Choi et al., 2017; Kim et al., 2017; Li et al., 2020a). The signals of identified WDR neurons were high-pass filtered (250 to 7500 Hz) and digitized (30 kHz; Digital Headstage Processor, Plexon, Dallas, TX, USA). The spikes were stored by OmniPlex Software (Plexon, Dallas, TX, USA) and analyzed with the Offline Sorter V4.0 (Plexon, Dallas, TX, USA) (Sun et al., 2022). Each raw trace of neuronal action potentials was obtained by NeuroExplorer software (Nex Technologies, Colorado Springs, CO, USA).

### 2.6 Depletion of serotonin or noradrenaline

**Figure 1:**
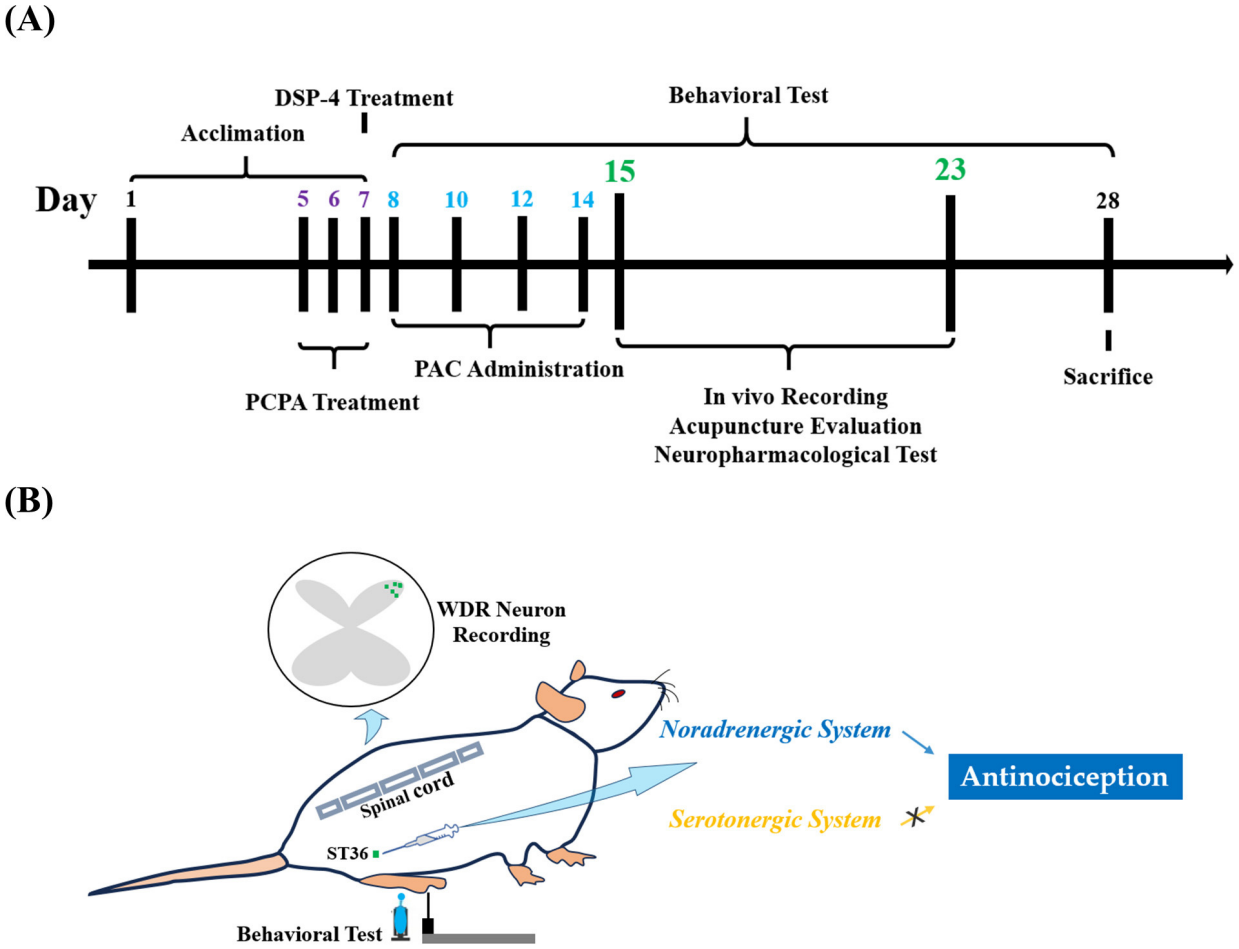
*In vivo* electrophysiological, neuropharmacological, and acupuncture tests were performed during days 15 to 23, when all three modalities of neuropathic signs were significant **(A)**. **(B)** Illustrates that extracellular single-unit recordings were obtained from the spinal dorsal horn. Behavioral examinations were run on the right hind limb before and after apitherapy at ipsilateral ST36. Analgesic properties of melittin require the specific recruitment of the endogenous noradrenergic system. **(B)** DSP-4, N-(2-Chloroethyl)-N-ethyl-2-bromobenzylamine hydrochloride; PCPA, Para-chlorophenylalanine; PAC, paclitaxel; WDR neuron, wide dynamic range neuron; ST36, Zusanli acupoint.

Pre-administrations with relevant agents were performed to decipher potential endogenous serotonergic or noradrenergic mechanisms of melittin on paclitaxel-induced neuropathy. Serotonin (5-HT) was depleted by three consecutive injections of Para-chlorophenylalanine (PCPA, 150 mg/kg/day, i.p.; days 5 to 7; Aladdin Scientific, Shanghai, China) (Li et al., 2019). Besides, to deplete noradrenaline (NA), rats were subjected to a single injection of N-(2-Chloroethyl)-N-ethyl-2-bromobenzylamine hydrochloride (DSP-4, 50 mg/kg, i.p.; day 7; Macklin Biochemical Technology Co., Ltd., Shanghai, China) (Li et al., 2019). The concentrations and schedules of DSP-4 and PCPA applications were precisely selected based on published data demonstrating that these regimens deplete 5-HT and NA in the central nervous system without influencing the onset of CIPN symptoms (Jaim-Etcheverry and Zieher, 1980; Maleki et al., 2005; Li et al., 2015, 2019). Figure 1 shows the experimental flowchart and schematic illustration.

### 2.7 Statistics

Animals were excluded if paclitaxel regimens induced chemotherapeutic poisoning (e.g., alopecia, motor dysfunction), if there was severe subcutaneous infection or swelling at the ST36 area of the right hind limbs after apitherapy treatments, or if death was caused by excessive anesthesia of urethane or massive dorsal horn hemorrhage during *in vivo* spinal recordings. No animals met these exclusion criteria for the current statistical analysis.

Statistical analysis was run by Prism v10.0 (GraphPad Software, La Jolla, USA). Statistical testing was performed with Two-way ANOVA, followed by Bonferroni's multiple comparison test or unpaired *t*-test. Data are presented as mean ± standard error of the mean (SEM). Statistical significance is defined as *P* < 0.05.

## 3 Results

### 3.1 Cold and mechanical peripheral neuropathy following paclitaxel regimens in rats

**Figure 2:**
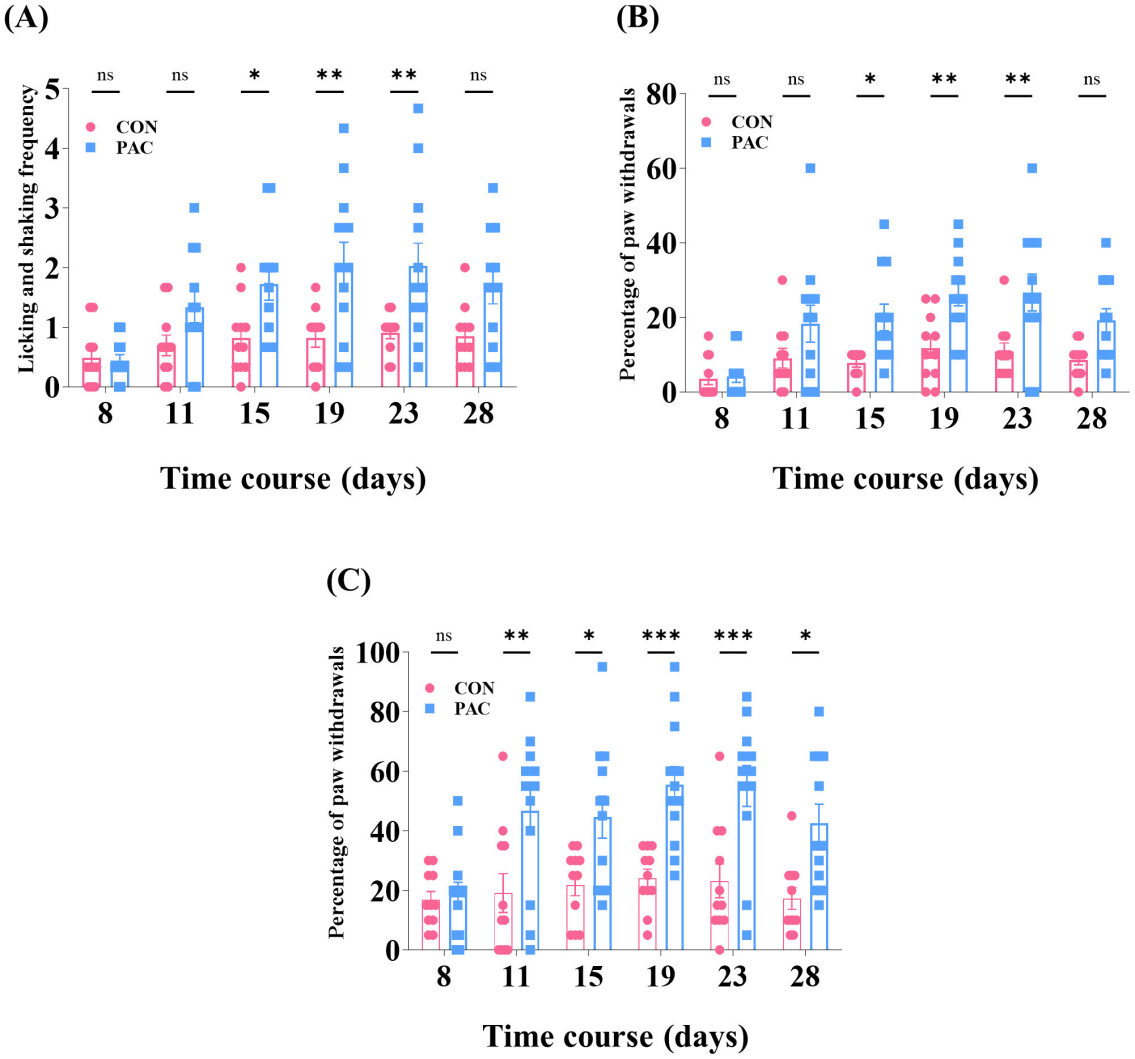
Progression of peripheral cold and mechanical hypersensitivities over time in the chemotherapy and control groups. Paclitaxel (PAC, *n* = 12) or vehicle (control, CON, *n* = 11) was dosed once daily on four alternate days (days 8, 10, 12, and 14). Withdrawal responsiveness was investigated just before the first dose of paclitaxel on day 8 and re-tested 5 times over the next 3 weeks (timeline: days 8, 11, 15, 19, 23, and 28). **(A)** Indicates the frequency of withdrawals exerted by 50 μL of acetone was counted over 30 s. Withdrawal frequencies of the right hind paw by the von Frey filament [VFF, with a bending force of 4 or 15 g, **(B, C)**] were expressed as a percentage value: (number of withdrawals × 100)/(total number of trials). Error bars represent the mean ± SEM; *p < 0.05, **p < 0.01, ***p < 0.001, vs. control; by Bonferroni *post-hoc* test after two-way ANOVA [**(A)**, *F*_(1, 126)_ = 32.07, *p* < 0.0001; **(B)**, *F*_(1, 126)_ = 34.42, *p* < 0.0001; **(C)**, *F*_(1, 126)_ = 50.75, *p* < 0.0001].

Four systemic administrations of paclitaxel (2 mg/kg/day, i.p.; days 8, 10, 12, and 14) exerted marked peripheral hypersensitivities in rats during a 3-week evaluation period. Before the initiation of chemotherapy, baseline withdrawal responsiveness to cutaneous stimuli was identical between groups in all behavioral trials on day 8 (all *p* > 0.05, Figure 2). In the cold allodynia test, significant hypersensitivity (assessed by using 50 μL of acetone) emerged on day 15, and it lasted for 9 consecutive days with increasing severity (*p* < 0.05, day 15; *p* < 0.01, days 19 to 23, Figure 2A). In addition, marked increments in brisk withdrawal in response to mechanical stimuli were validated in the chemotherapeutic group in contrast to the controls, indicating the establishment of significant mechanical allodynia from day 15 (*p* < 0.05), escalating during days 19 to 23 (*p* < 0.01, Figure 2B). Furthermore, mechanical hyperalgesia was also noticeably induced by paclitaxel, and its duration was the longest-lasting among the three neuropathic signs (*p* < 0.01, day 11; *p* < 0.001, days 19 to 23; *p* < 0.05, days 15 and 28, Figure 2C). Accordingly, the subsequent behavioral, electrophysiological, and neuropharmacological analyses were undertaken from days 15 to 23, the overlapping window for three modalities of neuropathic symptoms.

### 3.2 Ameliorative action of bvPLA2 and melittin against paclitaxel-induced peripheral neuropathy

**Figure 3:**
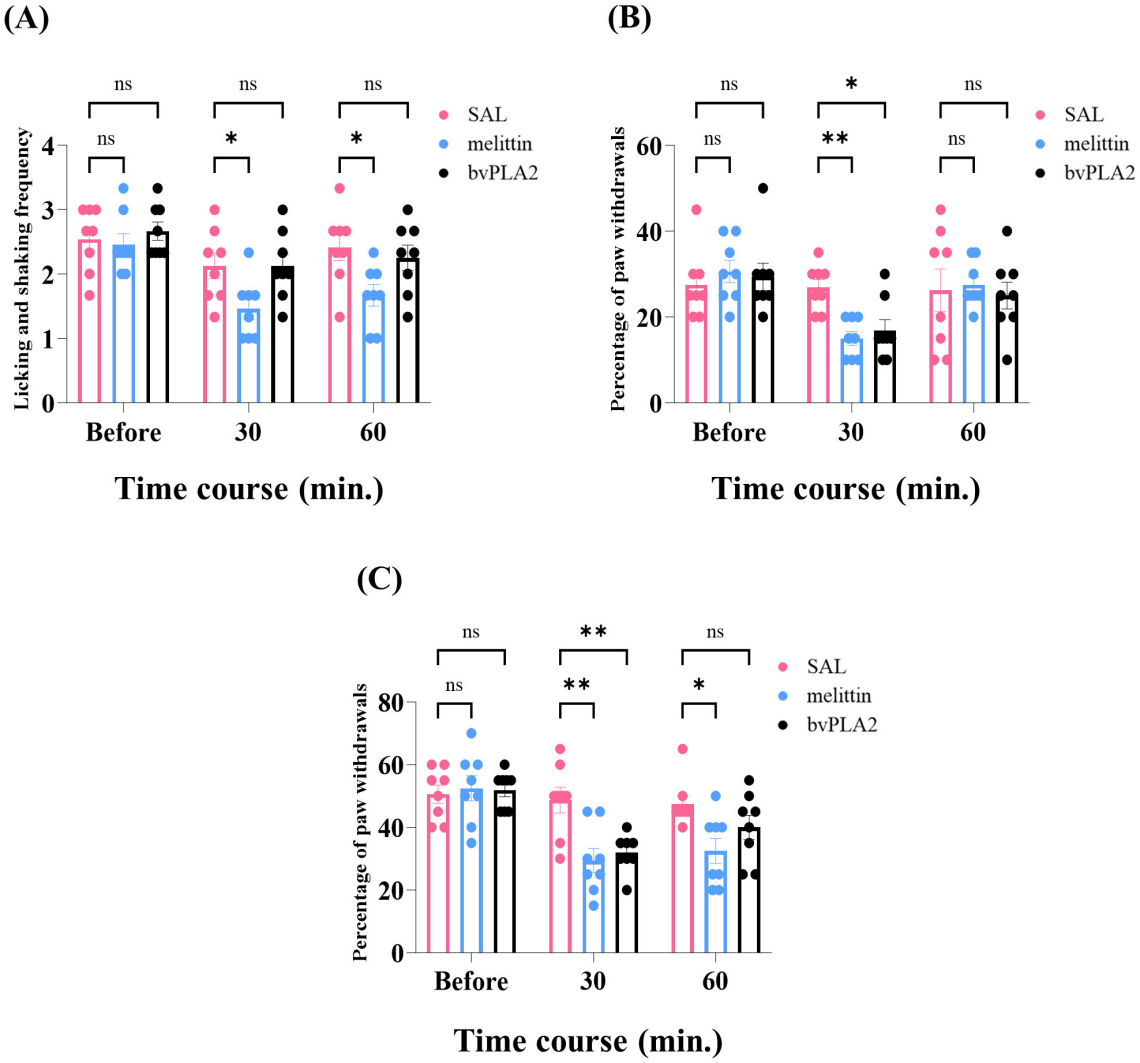
Temporal profile of the relieving effects of pharmacopuncture on paclitaxel-induced neuropathic states. Rats were subcutaneously dosed with melittin, bvPLA2, or saline (SAL, control) at ST36 (*n* = 8/group). Evaluations of neuropathic signs before acupuncture were re-done at 30-min intervals for 60 min post-treatment [timeline: before, 30, and 60, **(A–C)**]. Error bars represent the mean ± SEM; *p < 0.05, **p < 0.01, vs. control; by Bonferroni *post-hoc* test after two-way ANOVA [**(A)**, *F*_(21, 42)_ = 3.251, *p* = 0.0006; **(B)**, *F*_(21, 42)_ = 2.012, *p* = 0.0267; **(C)**, *F*_(21, 42)_ = 3.710, *p* = 0.0001]. bvPLA2, bee venom phospholipase A2.

To comparatively examine the analgesic properties of the main bioactive substances of bee venom, bvPLA2 (0.12 mg/kg), melittin (0.5 mg/kg), or saline (SAL, 50 μL, as control) were arbitrarily injected at ST36 of the right hind limb. Each group contained 8 rats exhibiting concurrent mechanical allodynia, hyperalgesia, and cold allodynia. Deteriorations of neuropathic symptoms were identified before acupoint maneuvers and re-tested at 30-min intervals for 60 min post-treatment (Figure 3). Compared to controls, cold hypersensitivity to the acetone cooling stimulus was not influenced after bvPLA2 therapy (*p* > 0.05, 30, and 60 min, Figure 3A). By contrast, bvPLA2 substantially eased the mechanical withdrawal responses of the ipsilateral hind paw to both 4 and 15 g VFF stimulus, which persisted up to 30 min, respectively (allodynia, *p* < 0.05; and hyperalgesia, *p* < 0.01, Figures 3B, C). Similarly, regardless of the forms of mechanical evaluations, potent decrements in total withdrawal percentages of the hind paw were shown in melittin-treated rats (allodynia, *p* < 0.01, 30 min; and hyperalgesia, *p* < 0.01, 30 min and *p* < 0.05, 60 min, Figures 3B, C). The acetone drop assay also identified anti-allodynic properties of melittin, spanning 60 min (*p* < 0.05, 30, and 60 min, Figure 3A). Based on the peripheral findings, since bvPLA2 showed limited alleviative effect on cold allodynia, we subsequently sought to explore alterations in neuronal hyperexcitability in the spinal dorsal horn following melittin application at ST36.

### 3.3 Activity of WDR neurons in the spinal dorsal horn could be enhanced after paclitaxel administration *in vivo*

**Figure 4:**
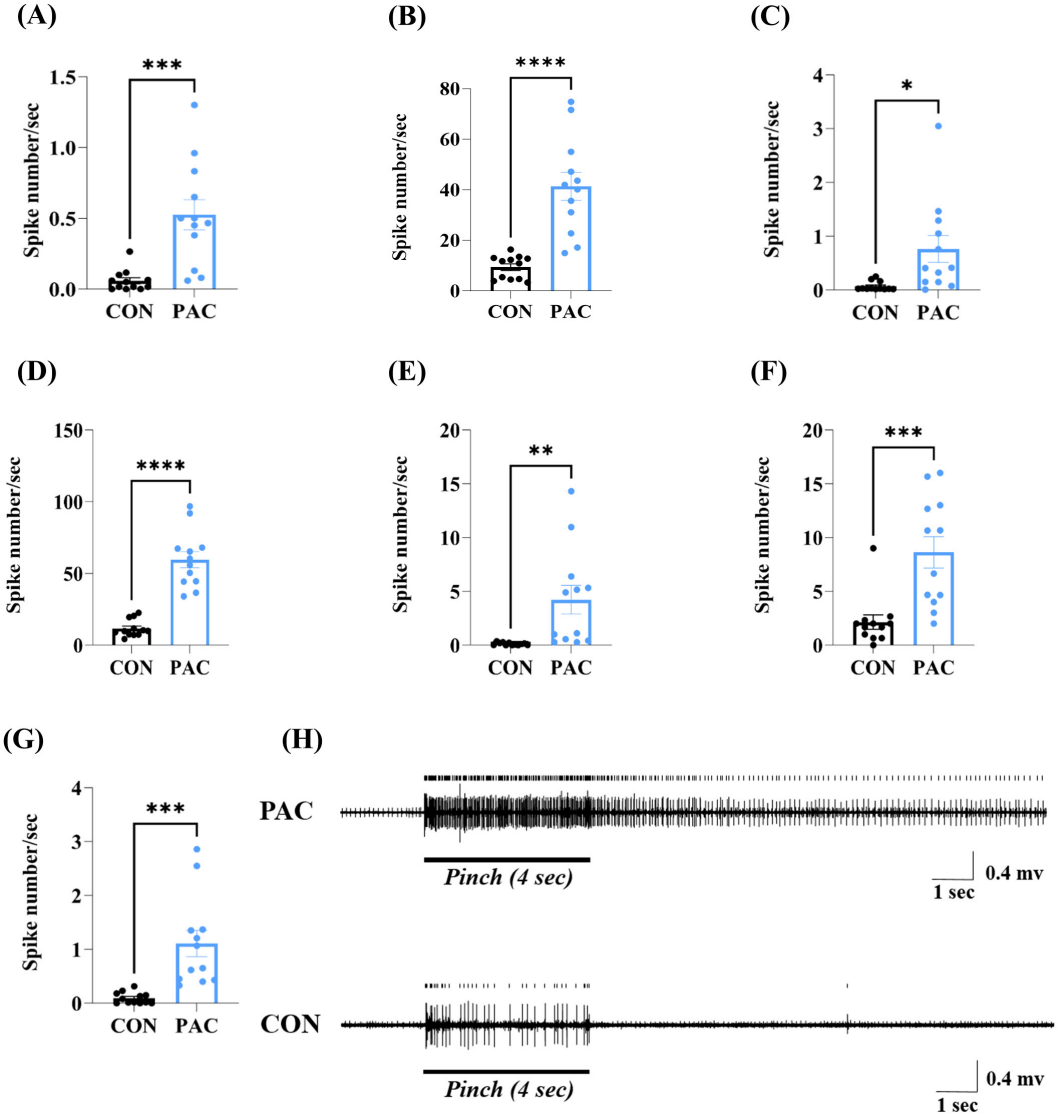
Paclitaxel-induced hyperactivity of WDR neurons in the spinal dorsal horn *in vivo*. Spontaneous action potential [0 to 60 s before stimulus, **(A)**], peripheral stimulus-evoked firing, and after-discharge that followed applications of brush, pinch, and acetone stimuli (after-discharge to mechanical: 0 to 60 s post-stimulus; after-discharge to cold: 3 to 63 s post-acetone application) were continuously recorded in the neuropathic and control groups [**(B–G)**, *n* = 12/group]. Animals received three cutaneous stimuli (brush, pinch, and acetone drop) in the peripheral receptive field restricted to the ipsilateral hind paw. Typical analog traces detected from spinal WDR neurons demonstrate responses to mechanical pinch stimuli, including acute response and after-discharge **(H)**. Error bars represent the mean ± SEM; *p < 0.05, **p < 0.01, ***p < 0.001, ****p < 0.0001; by unpaired *t*-test [**(A)**, *F*_(11, 11)_ = 23.56, *p* < 0.0001; **(B)**, *F*_(11, 11)_ = 17.05, *p* < 0.0001; **(C)**, *F*_(11, 11)_ = 107.5, *p* < 0.0001; **(D)**, *F*_(11, 11)_ = 11.45, *p* < 0.0003; **(E)**, *F*_(11, 11)_ = 1442, *p* < 0.0001; **(F)**, *F*_(11, 11)_ = 4.82, *p* = 0.0148; **(G)**, *F*_(11, 11)_ = 61.74, *p* < 0.0001]. PAC, paclitaxel; CON, control.

Systemic paclitaxel regimens have induced marked peripheral cold and mechanical disturbance (Figure 2). Next, we employed *in vivo* extracellular recording techniques to examine spontaneous and peripheral stimulus-elicited action potentials of spinal WDR neurons in paclitaxel-induced neuropathic states. In the CIPN group, the spontaneous mean firing frequencies (events/seconds) of WDR neurons were higher than those in controls (0.5258 ± 0.1067 vs. 0.0591 ± 0.0219 spikes/s, *p* < 0.001, Figure 4A). The receptive field of the isolated WDR neuron was stimulated in the following order: brush, pinch, and acetone cooling. Compared to vehicle-injected rats, remarkable increments in the mean discharge rate during the peripheral mechanical (dynamic brush and pinch) stimuli and after-discharge (AD) frequency of WDR neurons that followed applications of cutaneous mechanical stimuli were quantified in rats dosed with paclitaxel (brush, 9.371 ± 1.338 vs. 41.35 ± 5.525 spikes/s, *p* < 0.0001; AD to brush, 0.06983 ± 0.02421 vs. 0.7623 ± 0.251 spikes/s, *p* < 0.05; pinch, 11.67 ± 1.694 vs. 59.63 ± 5.734 spikes/s, *p* < 0.0001; AD to pinch, 0.1209 ± 0.03525 vs. 4.226 ± 1.329 spikes/s, *p* < 0.01, Figures 4B–E). Likewise, acute discharge rate and AD frequency of WDR neurons induced by applications of evaporative cooling stimulation (50 μL of acetone) were elevated in neuropathic individuals (acetone, 2.139 ± 0.6633 vs. 8.639 ± 1.456 spikes/s, *p* < 0.001; AD to acetone, 0.09547 ± 0.03080 vs. 1.107 ± 0.2420 spikes/s, *p* < 0.001, Figures 4F, G). This hyperactivity of spinal WDR neurons (i.e., increased spontaneous discharges, amplified acute responses, and elevated ADs) correlated with the peripheral neuropathic signs established following paclitaxel regimens (Figure 2). Figure 4H shows typical analog traces of the WDR neuron's discharge responses to peripheral pinch stimuli.

### 3.4 Melittin at ST36 dampened the hyperexcitation of spinal WDR neurons within the paclitaxel-induced neuropathic condition

**Figure 5:**
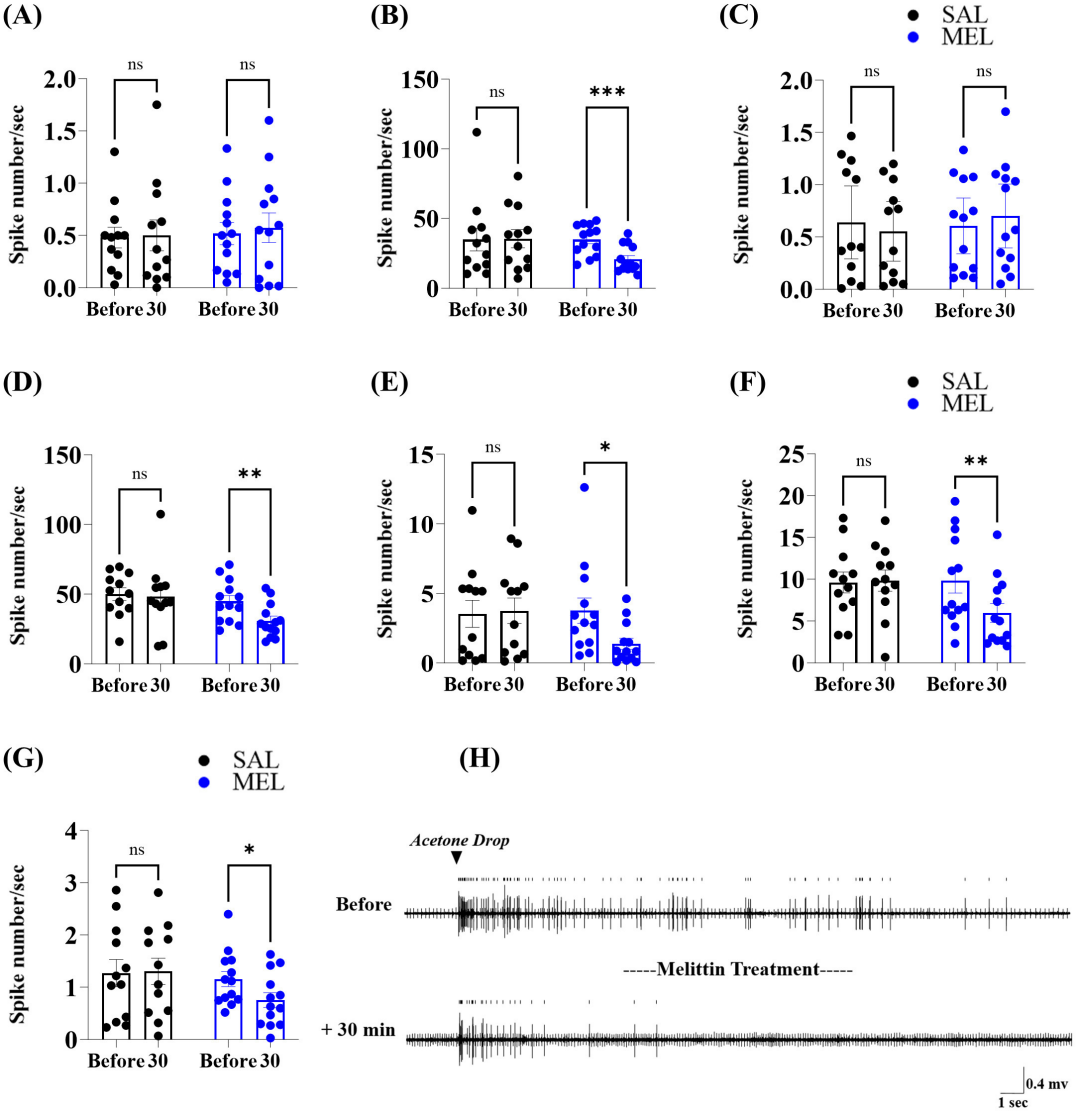
Melittin suppressed the spinal neuronal activity of the neuropathic rats. Discharge waveforms of dorsal horn WDR neurons were continuously recorded before treatment with melittin (MEL, *n* = 13) or saline (SAL, control, *n* = 12) at ipsilateral ST36 and 30 min post-therapy [timeline: before and 30; **(A–G)**]. Rats were subjected to three cutaneous stimuli (brush, pinch, and acetone) in the peripheral receptive field restricted to the ipsilateral hind paw. The action potentials of the WDR neuron include spontaneous firing **(A)**, acute response, and after-discharge to brush **(B, C)**, pinch **(D, E)**, and acetone stimuli **(F, G)**. Typical analog traces detected from spinal WDR neurons indicate acetone stimulus-elicited action potentials (acute response: 0 to 3 s, after-discharge: 3 to 63 s), mitigated after apitherapy at ipsilateral ST36 **(H)**. Error bars represent the mean ± SEM; *p < 0.05, **p < 0.01, ***p < 0.001, vs. Before; by Bonferroni *post-hoc* test after two-way ANOVA [**(A)**, *F*_(1, 23)_ = 0.02978, *p* = 0.8645; **(B)**, *F*_(1, 23)_ = 9.611, *p* = 0.0050; **(C)**, *F*_(1, 23)_ = 1.395, *p* = 0.2496; **(D)**, *F*_(1, 23)_ = 6.567, *p* = 0.0174; **(E)**, *F*_(1, 23)_ = 5.070, *p* = 0.0342; **(F)**, *F*_(1, 23)_ = 5.641, *p* = 0.0263; **(G)**, *F*_(1, 23)_ = 3.257, *p* = 0.0842].

To decipher whether melittin administration abolishes the hyperactivity of spinal WDR neurons in rats with neuropathy, we quantified the frequencies of action potentials before melittin treatment and re-examined 30 min post-apitherapy. No marked suppression of spontaneous firing frequency was observed following melittin administration (0.5180 ± 0.1053 vs. 0.5743 ± 0.1410 spikes/s, *p* > 0.05, Figure 5A). Instead, melittin significantly attenuated stimulus-evoked acute responses: dynamic brush (34.90 ± 3.023 vs. 20.80 ± 2.665 spikes/s, *p* < 0.001, Figure 5B), pinch (44.89 ± 4.150 vs. 30.95 ± 3.371 spikes/s, *p* < 0.01, Figure 5D), and acetone cooling (9.846 ± 1.504 vs. 5.974 ± 1.127 spikes/s, *p* < 0.05, Figure 5F), responses were all markedly reduced. Additionally, brush-evoked AD remained unaffected by melittin (0.6057 ± 0.1225 vs. 0.7008 ± 0.1397 spikes/s, *p* > 0.05, Figure 5C), while AD to pinch and cooling stimuli was decreased by melittin (AD to pinch, 3.758 ± 0.9106 vs. 1.366 ± 0.4034 spikes/s; AD to acetone, 1.157 ± 0.1451 vs. 0.7534 ± 0.1415 spikes/s, all *p* < 0.05, Figures 5E, G). Control rats treated with saline (SAL, 50 μL) at ST36 showed no significant changes in spontaneous firing (0.4803 ± 0.0991 vs. 0.5014 ± 0.1480 spikes/s, *p* > 0.05, Figure 5A), acute response (brush, 34.89 ± 8.134 vs. 35.54 ± 6.459 spikes/s; pinch, 50.01 ± 4.528 vs. 48.21 ± 6.977 spikes/s; acetone, 9.610 ± 1.252 vs. 9.833 ± 1.250 spikes/s, all *p* > 0.05, Figures 5B, D, F), and ADs (AD to brush, 0.6385 ± 0.1583 vs. 0.5540 ± 0.1302 spikes/s; AD to pinch, 3.532 ± 0.9781 vs. 3.747 ± 0.9202 spikes/s; AD to acetone, 1.273 ± 0.2603 vs. 1.304 ± 0.2502 spikes/s, all *p* > 0.05, Figures 5C, E, G) of WDR neurons. A representative raw trace illustrating the decrease in WDR neurons' discharges to the acetone drop stimulus 30 min after melittin therapy is shown in Figure 5H. Our electrophysiological data indicated that noticeable attenuations of spinal neuronal sensitizations in the dorsal horn following apitherapy at ipsilateral ST36 correlated with the melittin-induced alleviative action mentioned above (Figure 3), underscoring spinal modulation underlying its analgesia against paclitaxel-induced neuropathic ailments.

### 3.5 Noradrenergic or serotonergic roles in the effects of melittin against paclitaxel-induced neuropathy in rats

**Figure 6:**
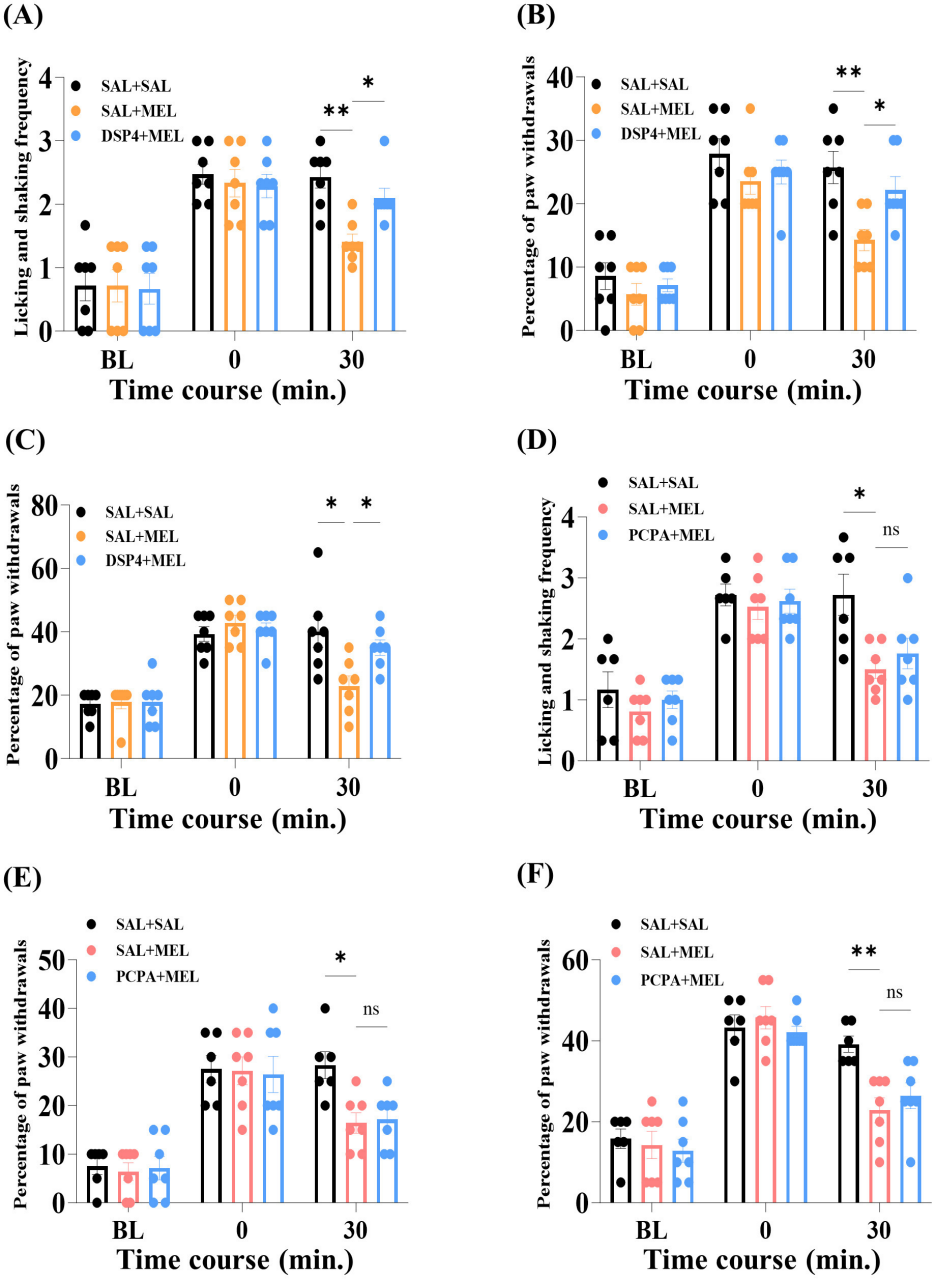
Effects of depletion of noradrenaline and serotonin on the melittin-induced analgesia. Serotonin was depleted by three consecutive injections of PCPA (150 mg/kg/day, i.p., *n* = 6). To examine the involvement of noradrenaline, a single injection of DSP-4 (50 mg/kg, i.p., *n* = 7) was performed before chemotherapy. Baseline (BL) sensitivities were measured on day 8. The levels of CIPN were tested just before (0) and 30 min post-apitherapy [30, **(A–F)**]. Error bars represent the mean ± SEM; *p < 0.05, **p < 0.01, vs. the SAL + MEL group; by Bonferroni *post-hoc* test after two-way ANOVA [**(A)**, *F*_(4, 36)_ = 2.070, *p* = 0.1052; **(B)**, *F*_(4, 36)_ = 3.514, *p* = 0.0160; **(C)**, *F*_(4, 36)_ = 4.519, *p* = 0.0046; **(D)**, *F*_(4, 34)_ = 3.316, *p* = 0.0214; **(E)**, *F*_(4, 34)_ = 3.733, *p* = 0.0127; **(F)**, *F*_(4, 34)_ = 3.462, *p* = 0.0178]. SAL, saline; MEL, melittin; DSP-4, N-(2-Chloroethyl)-N-ethyl-2-bromobenzylamine hydrochloride; PCPA, Para-chlorophenylalanine.

Systemic depletions of relevant neurotransmitters were performed before acupuncture treatments to examine the involvement of noradrenergic or serotonergic pathways in the alleviative action of melittin. In this context, rats were randomly dosed with DSP-4 (50 mg/kg, day 7), PCPA (150 mg/kg/day, days 5 to 7), or SAL (controls). We investigated the onset of cold (Figures 6A, D) and mechanical allodynia (Figures 6B, E) and mechanical hyperalgesia (Figures 6C, F) from days 15 to 23, and neuropathic signs were reassessed 30 min after melittin application. Independent of the type of trials, melittin failed to eliminate peripheral hypersensitivities following the depletion of NA 30 min after apitherapy (SAL + MEL vs. DSP-4 + MEL, *p* < 0.05, Figures 6A–C). On the other hand, 5-HT depletion by PCPA had no significant influence on the effects of melittin, both in acetone and VFF trials (SAL + MEL vs. PCPA + MEL, *p* > 0.05, Figures 6D–F). Overall, the protective action of melittin against paclitaxel-induced peripheral neuropathy requires the involvement of the endogenous noradrenergic pathway in rats.

## 4 Discussion

Compared with other cytostatic strategies, a paclitaxel-containing chemotherapeutic regimen is more efficacious against an array of lung, pancreatic, and gynecologic solid malignancies (e.g., fallopian tube cancer and endometrial cancer), among others, which makes it a cornerstone option for a large cohort of cancer survivors (Tasmuth et al., 2002; Beh et al., 2019; Kawashiri et al., 2021; Tsai et al., 2021; Yu et al., 2022). Despite this, dose-dependent neuropathic impairments caused by paclitaxel occur more frequently than those induced by other taxane-based regimens (e.g., docetaxel) to some extent (Tsai et al., 2021). As would be expected, such daunting side effects tremendously compromise clinical adherence for those last-resort survivors (Boyette-Davis et al., 2015). Beyond doubt, it is crucial to implement rational remedies for combating paclitaxel-induced neuropathic ailments in oncological care (Boyette-Davis et al., 2018; Zajaczkowska et al., 2019).

The signs of peripheral mechanical allodynia, hyperalgesia, and cold allodynia are conventionally utilized as read-outs of the manifestation of CIPN and the therapeutic properties of analgesics in rodent models (Polomano et al., 2001; Flatters et al., 2006; Siau et al., 2006). Our study demonstrated that systemic paclitaxel regimens produce tri-modal peripheral hypersensitivities (Figure 2). Besides, ST36 is located below the knee joint and near the peripheral ascending nerve pathways from the extremities (Yin et al., 2008; Choi et al., 2019). Several articles have chosen this acupoint to dissect the analgesic capacity of acupuncture-based maneuvers (Kim et al., 2005; Lu et al., 2016; Zeng et al., 2018). Recently, we have identified the location-specific analgesic profile of bee venom (ST36 acupoint vs. non-acupoint) against CIPN caused by paclitaxel (Li et al., 2020b). The amounts of melittin and bvPLA2 contained in the dry bee venom were 50 and 12%. Building upon earlier findings of the reliable analgesia of BVA (1.0 mg/kg) toward CIPN (Kim et al., 2016; Li et al., 2020b), in this article, we further explored the component-specific analgesic profile of bee venom. Of note, there was a difference between the analgesic actions of 0.5 mg/kg of melittin and 0.12 mg/kg of bvPLA2 against paclitaxel-induced neuropathy. We found that the s.c. stimulation of ST36 with melittin but not with bvPLA2 noticeably dampened cold disturbance (Figure 3A). These results and a few pieces of previous evidence indicate that melittin, compared with other bee venom ingredients, possesses broader therapeutic effects on CIPN (Choi et al., 2019; Li et al., 2020a).

Our electrophysiological data revealed a marked reinforcement of spontaneous activity within spinal WDR neurons under paclitaxel-induced neuropathic states (Figure 4A). A few papers have demonstrated that WDR neurons exhibit enhanced spontaneous discharge in a host of pain conditions (McGaraughty et al., 2018; Zain and Bonin, 2019). Such elevated spontaneous neuronal signals have been established as one of the hallmarks of central sensitization under painful conditions (Latremoliere and Woolf, 2009; Zhang et al., 2024). In this study, the application of melittin at ST36 had limited effects on the spontaneous firing of WDR neurons (Figure 5A), suggesting that this pharmacoacupuncture failed to completely counteract the paclitaxel-dependent spinal sensitization. Consistent with our findings, some pharmacological agents (e.g., a Cav 3.2-specific blocker) could modulate mainly evoked but not spontaneous activity of WDR neurons (McGaraughty et al., 2018).

It is known that peripheral excitatory and inhibitory inputs converge at a node constituted by spinal pain relay neurons (Latremoliere and Woolf, 2009; Baron et al., 2010; Todd, 2010; Gilron et al., 2013). Our investigation provides the first evidence of WDR neuronal responses to peripheral evaporative cooling stimuli in the context of paclitaxel-induced neuropathic states (Figures 4, 5). Remarkable analgesia generally coincides with a decrement in stimuli-evoked acute response or ADs of WDR neurons in the central nervous system (Chen et al., 2005; McGaraughty et al., 2018; Yamada et al., 2018; Zain and Bonin, 2019; Zhang et al., 2024). We determined that 0.5 mg/kg of melittin had broad-spectrum inhibitory actions on acute discharge during peripheral stimuli, with specific inhibition of ADs that were observed selectively in pinch and acetone stimuli, while showing negligible effects on brush-evoked ADs (Figure 5). One explanation for this difference may stem from distinct routes of sensory signaling, where transmissions of cutaneous cold and nociceptive mechanical information predominantly depend on C afferent fibers, while innocuous tactile signals are conveyed by both A and C fibers (Pertovaara, 1993; Millan, 2002; Reddi et al., 2013; Li et al., 2019). A second plausible reason is that spinal neuronal firing during mechanical stimuli is mainly associated with direct activation of primary afferent fibers, whereas ADs to brush stimuli originate from the inherent plateau potentials of pain-relay neurons (Yoshimura and Jessell, 1989; Morisset and Nagy, 1998; Furue et al., 1999). Consistent with electrophysiological findings, our behavioral test demonstrated that stimulation of ST36 with melittin reduced withdrawal responses to acetone and 15 g mechanical stimuli for 60 min (Figures 3A, C), while only alleviating 4 g bending force-induced withdrawals for 30 min (Figure 3B). Collectively, our results show that the s.c. injection of melittin at ipsilateral ST36 could have more pronounced and longer-lasting analgesic effects on cold allodynia and mechanical hyperalgesia than on mechanical allodynia in paclitaxel-treated rats.

In our neuropharmacological investigation, the i.p. pre-depletion of NA, but not 5-HT, reversed the analgesic action of melittin on the paclitaxel-induced peripheral neuropathic state (Figure 6). This suggests that the underlying mechanism of its benefits involves the specific recruitment of the endogenous noradrenergic inhibitory system, a finding consistent with previous results in oxaliplatin-induced allodynic behavior (Choi et al., 2019). The endogenous noradrenergic system uses NA as the main neurotransmitter to activate adrenoceptors (Millan, 2002), thereby inhibiting nociceptive signals and ultimately inducing pain relief (Yoshimura and Furue, 2006). According to our previous practice, it has been demonstrated that local silencing of the locus coeruleus (LC, the main source of NA) by lidocaine could reverse BVA-induced analgesia in vincristine-dependent neuropathy (Li et al., 2020a). Similarly, activation of adrenoceptors by NA can modulate the firing frequency of WDR neurons in CIPN models (Choi et al., 2017). These mechanisms may also be the reason why serotonin-noradrenaline reuptake inhibitors (SNRIs), a class of antidepressants, exhibit analgesic efficacy in clinical patients with CIPN symptoms (Hershman et al., 2014). Notably, as a representative SNRI, the i.p. administration of duloxetine (30 mg/kg) has been revealed to protect rodents from oxaliplatin-induced hyperactivity of dorsal horn neurons (Kim et al., 2017). However, in our earlier study, both serotonergic and noradrenergic mechanisms modulated the pain relief effect of other therapies in the mechanical VFF assay (Li et al., 2019), which was somewhat different from the current results. We hypothesized that differences in analgesic interventions and antineoplastic regimens could partially underlie this outcome (melittin pharmacoacupuncture at ST36 vs. i.p. venlafaxine treatment, and multiple paclitaxel treatments vs. single oxaliplatin administration).

One limitation of the current study is that it focused on the analgesic mechanism only in male rats. For future research, standardized practices, such as whether melittin or bvPLA2 therapy exerts significant therapeutic properties in paclitaxel-induced neuropathic states and spinal hyperactivity in female animals, are required. Growing *in vitro* studies have identified that melittin suppresses the growth of leukemia, lung, and ovarian cancer cells without potentially affecting non-cancerous cells (Hait et al., 1985; Zhu et al., 1991; Gajski et al., 2024). These findings are worthy of note since the ideal strategy involves effectively mitigating the devastating CIPN without compromising the outcome of chemotherapy (Boyette-Davis et al., 2018). Consequently, robust trials with standardized methodologies are called for to determine the synergistic life-prolonging potential of melittin in combination with existing antineoplastic agents.

## 5 Conclusion

We confirmed that systemic paclitaxel regimens could induce peripheral cold and mechanical hypersensitivity (allodynia and hyperalgesia) in the hind paw of male rats. Peripheral neuropathic pain elicited by paclitaxel correlated with spontaneous and stimulus-evoked neuronal hyperexcitability in the spinal dorsal horn. A marked decrease in aberrant firings of WDR neurons can appear in the dorsal horn ipsilateral to the s.c. application of 0.5 mg/kg of melittin at ST36. These analgesic properties of melittin require the specific recruitment of the endogenous noradrenergic pathways. As more cancer patients receive paclitaxel-containing regimens, our findings could provide opportunities for developing reliable melittin-based analgesic strategies to address paclitaxel-induced peripheral neuropathic complications.

## Data Availability

The raw data supporting the conclusions of this article will be made available by the authors, without undue reservation.
